# Contrasting associations between microbiota respiratory strategies and seafloor macrofauna

**DOI:** 10.1093/ismeco/ycag230

**Published:** 2026-08-13

**Authors:** Knut Rudi, Sanna Majaneva, Jessica Louise Ray, Maud Sundt, Julie Martin, Inga Leena Angell, Stian Nylund, Gro Harlaug Refseth, Nigel Keeley, Lars-Gustav Snipen

**Affiliations:** Department of Chemistry, Biotechnology and Food Science, Norwegian University of Life Sciences, 1433 Ås, Norway; Akvaplan-niva AS, 7010 Trondheim, Norway; Aqua Kompetanse AS, 5006 Bergen, Norway; STIM AS, Lofoten, 9008 Tromsø, Norway; Department of Chemistry, Biotechnology and Food Science, Norwegian University of Life Sciences, 1433 Ås, Norway; Department of Chemistry, Biotechnology and Food Science, Norwegian University of Life Sciences, 1433 Ås, Norway; STIM AS, Lofoten, 9008 Tromsø, Norway; Akvaplan-niva AS, 7010 Trondheim, Norway; Institute of Marine Research, 9007 Tromsø, Norway; Department of Chemistry, Biotechnology and Food Science, Norwegian University of Life Sciences, 1433 Ås, Norway

**Keywords:** seafloor, microbiota, macrofauna, metagenome, respiration

## Abstract

Microbe–macrofauna associations are central to coastal benthic ecosystems, yet the microbial functions that underpin these remain poorly understood. To address functional microbe–macrofauna associations, we analyzed 245 seafloor samples from Norwegian and Icelandic coastal zones, encompassing 1204 macrofaunal taxa, 121 543 zero-radius operational taxonomic units, and 302 high-quality metagenome-assembled genomes (MAGs). We first analyzed potential confounding factors and found that macrofauna showed the strongest association with a north–south geographic gradient, while overall there was a concordance for metadata associations. We found that macrofaunal assemblages were positively correlated with either a Bacteroidota-enriched cluster of MAGs (“BA”; *n* = 178 MAGs) or a cluster enriched in Actinomycetota and Pseudomonadota (“AP”; *n* = 124 MAGs). Functionally, the AP cluster was enriched in oxidases, cytochrome c-based respiratory processes, and fatty-acid β-oxidation. In contrast, the BA cluster showed enrichment in hydrolases, polysaccharide-degradation functions, and microaerobic/anaerobic respiration via cytochrome bd. Macrofaunal taxa associated with BA tended to have higher tolerance values for anthropogenic disturbance than those associated with AP, although this association was weak. Taken together, these results identify contrasting microbial respiratory strategies that co-vary with macrofaunal assemblages, while further experimental work is required to establish directionality and mechanism.

## Introduction

Coastal regions represent some of the most productive, yet vulnerable, marine environments on Earth [1]. These areas face intense anthropogenic pressure through pollution, resource exploitation, and the effects of climate change [2]. It has been known for decades that there is a gradient in macrofauna community composition that reflects the level of organic enrichment due to anthropogenic activity [3]. More recently, similar associations have been determined between organic enrichment and the microbiota [4]. Although the macrofauna and microbiota show similar associations with organic enrichment, we still have limited knowledge about potential mechanistic interactions [5] and how such interactions may impact ecosystem structure and function. Several open questions remain with respect to the directionality of these associations, including whether the microbiota structures the macrofauna, whether the macrofauna structures the microbiota, whether both respond independently to shared environmental gradients such as organic enrichment and redox conditions, or whether feedbacks operate among these processes.

High diversity, with most microorganisms being non-culturable, presents a challenge for analyses of seafloor ecology [6]. Although some functional inferences can be made from 16S rRNA gene sequencing, taxonomic and functional decoupling makes the inference difficult [7]. Furthermore, common 16S rRNA gene sequence denoising tools may also underestimate diversity in marine sediments, as true diversity can be treated as noise [8]. To improve these limitations, shotgun metagenomic sequencing may facilitate the deciphering of potential mechanistic associations in marine ecosystems without the need for functional inference. A recent study of marine metagenomes, however, identified a total of only 86 seafloor samples that have been shotgun-sequenced from the global oceans [9]. This stands in stark contrast to human [10] and terrestrial systems [11], where tens of thousands of shotgun-sequenced metagenomes are already available.

The aim of the current work was therefore to address underlying structures in the association between environmental microbes and macrofauna in soft-bottom seafloor ecosystems along the Norwegian and Icelandic coasts. This was done using a recent dataset containing information about metadata and the macrofauna community composition, in addition to 16S rRNA and shotgun metagenome sequences [5, 12].

**Figure 1:**
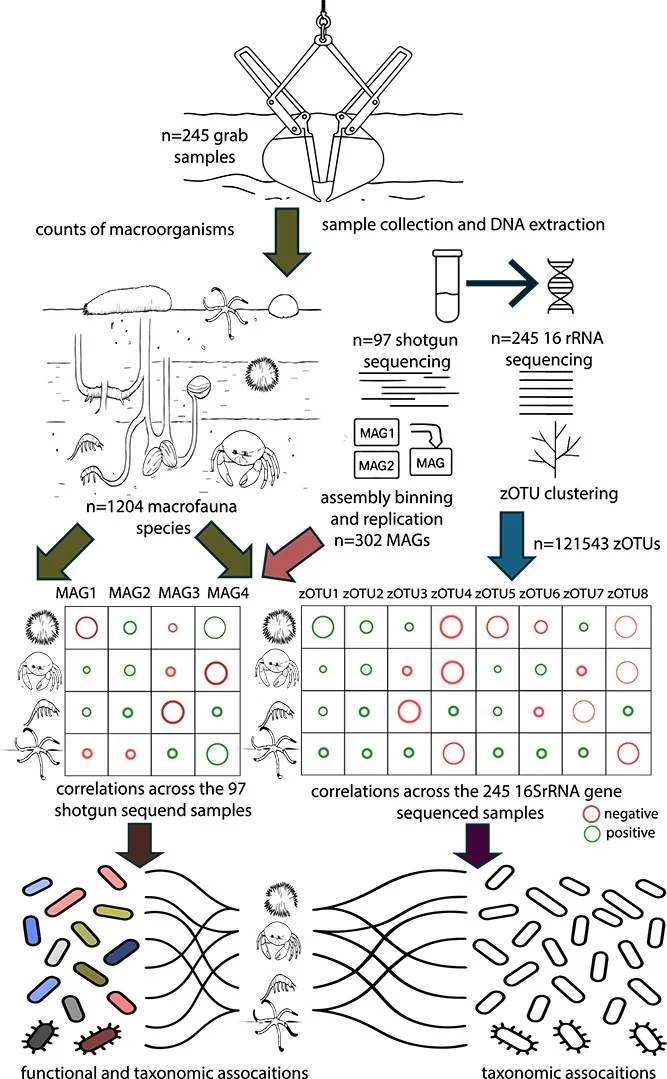
Overview of the experimental setup. A total of 245 grab samples were collected along the Norwegian and Icelandic coast. Macrofauna community composition was morphologically identified, and DNA was extracted from sub-sample for all the samples. All DNA samples were 16S rRNA gene sequenced, while a subset of 97 samples was also shotgun sequenced. Correlation analysis was performed for macrofauna to shotgun data through MAGs, and to the 16S rRNA gene data through zOTUs, respectively. The size of the circles inside the correlation matrices presents the magnitude of correlation coefficient between each macrofaunal species (rows) and each MAG or zOTU (columns), while the color represent the direction (red, negative and green, positive). In addition, functional and taxonomic associations were derived from the MAG and zOTU correlations.

An outline of the experimental setup is presented in Fig. 1.

## Materials and methods

### Metadata

**Figure 2:**
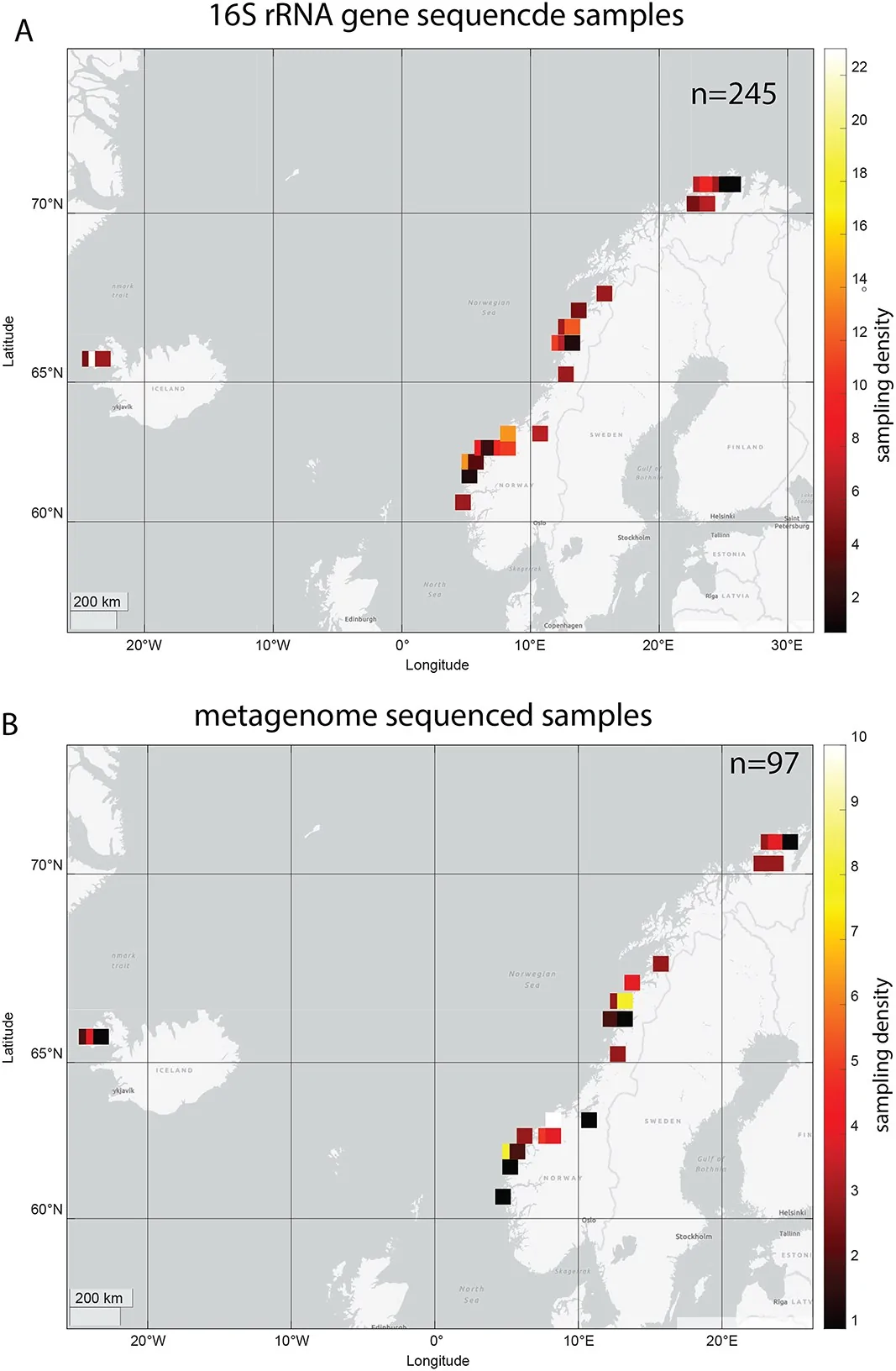
Geographical location for the included samples. (A) Location of the samples used for the 16S rRNA gene comparison, and (B) location of samples used for the shotgun sequencing.

We analyzed the association between macrofauna community composition and 16S rRNA gene zero-radius operational taxonomic units (zOTUs) for the full set of 245 samples, while the association between macrofauna and metagenome-assembled genomes (MAGs) were analyzed for a subset of 97 samples. The metadata for the zOTU dataset is provided in Supplementary Table S1, while the metadata for the MAG-based dataset is provided in Supplementary Table S2. The geographical distributions of the samples included in the two datasets are shown in Fig. 2.

### Macrofauna counts

The macrofauna data were derived according to ISO 16665:2014. Briefly, this involves counting animals with body size >1 mm from a 0.1 m^2^ Van Veen grab after sieving through a 1 mm sieve. The animals are manually identified to the lowest practically possible taxonomic level using a stereo microscope and standard taxonomic keys, with taxonomic names harmonized against the World Register of Marine Species (WoRMS) database. The total counts per sample are presented in Supplementary Tables S1 and S2, corresponding to the 16S rRNA gene and shotgun sequencing datasets, respectively.

### Shotgun metagenomic and 16S rRNA gene sequencing

We re-analyzed the raw 16S rRNA and shotgun sequence data available under accession number PRJNA1128851 in the Sequence Read Archive (SRA). These represent sediment samples collected along the Norwegian and Icelandic coast [5]. The 16S rRNA gene sequence data were processed using the algorithm implemented in USEARCH [13] for determining zOTUs, as described by Nilsen *et al*. [8]. The percentage of each zOTU in each sample is presented in Supplementary Table S3.

The MAG data were assembled, annotated, and the relative abundance determined as described previously [12], with functional assignments directly inferred from the provided Kyoto Encyclopedia of Genes and Genomes (KEGG) Orthologs (KOs). The KO content for each MAG is presented in Supplementary Table S4, while the relative abundance of the MAGs in the samples is presented in Supplementary Table S5.

### Dataset correlations

Benjamini–Hochberg false discovery rate (FDR) corrections were used where statistical significances were reported for more than one *P*-value. Using Spearman correlation, we correlated macrofauna counts separately with zOTU relative abundances and MAG coverages. This produced two datasets: one of zOTU-macrofauna correlations and one of MAG-macrofauna correlations. Each matrix containing the respective correlations was then mean-centered and reduced using principal component analysis (PCA). No additional variance scaling was applied because all input values were correlations on a common scale.

To test whether macrofauna showed similar correlation patterns with both zOTUs and MAGs, we analyzed the first principal component (PC1) scores for the macrofauna from each dataset using Spearman correlation.

Associations between the metadata variables, macrofauna, zOTUs, and MAGs were also evaluated using Spearman rank correlations. PCAs were performed for each of the correlation matrices, with metadata as loadings and macrofauna, zOTUs, and MAGs as the respective PCA scores. Loadings for the first PC from each PCA were used to determine the concordance in the association of the metadata with macrofauna, zOTUs, and MAGs.

We also evaluated the internal correlation structure among the selected metadata variables through a Spearman correlation matrix. Principal coordinate analysis (PCoA) was then performed on this matrix using classical multidimensional scaling. Eigenvalues from the ordination were used to calculate the percentage of variance explained by each PCoA axis, based on the sum of positive eigenvalues.

### Functional associations

The first PC for the scores (macrofauna counts) and loadings (MAG coverage) from the PCA model for the macrofauna and MAG correlations were assessed with respect to bimodality using Silverman’s test [14]. This method identifies the minimum smoothing bandwidth required for a distribution to appear unimodal or have no more than a specified number of modes, with subsequent bootstrap resampling to test whether the observed multimodality is greater than expected by chance.

The MAG dataset was then divided into two groups according to the sign of the loading for the first PC. To evaluate the robustness of this grouping, we progressively reduced each group by excluding MAGs with loadings closest to zero, separately for the positive- and negative-loading groups. The loading groups were evaluated by summarizing MAG relative abundance profiles according to model-loading direction and magnitude. For thresholds ranging from 0 to 0.10 in increments of 0.01, variables with loadings below the negative threshold and above the positive threshold were identified separately. For each threshold, the corresponding MAG relative abundance values were summed per sample, generating negative- and positive-loading summary matrices. Pairwise relationships among the first 9 threshold-derived summaries were inspected using scatterplot matrices, with histogram distributions displayed using 20 bins.

Functional differences between the two MAG groups were assessed using the frequency of KOs. For each KO, we compared its occurrence between the positive- and negative-loading MAG groups. A first-order polynomial was fitted to the data using ordinary least-squares linear regression. Differences in KO abundance between groups were then evaluated by calculating, for each KO, the signed perpendicular distance from the fitted regression line, capturing both the magnitude and direction of deviation from the overall abundance relationship. Positive values indicate points lying below the fitted line, whereas negative values indicate points lying above the fitted line. KOs showing the largest signed distances between the two groups were interpreted as the main functional contributors distinguishing MAGs associated positively or negatively with the groups defined by the sign of the PC1 loading.

### Software and data processing tools

Statistical analyses were performed in MATLAB (MathWorks Inc., Massachusetts) using the Statistics and Machine Learning Toolbox, along with the PLS Toolbox (Eigenvector Inc., Washington). The 16S rRNA gene processing was done using the scripts provided in MiDiv-lab bioinformatics (https://github.com/larssnip/midiv), while the processing of the shotgun data was done using the scripts provided in MAGmachine (https://arken.nmbu.no/∼larssn/MiDiv/README_metagenomes.html). Macrofauna tolerance values were extracted from the AMBI database (https://ambi.azti.es/), while taxonomic information was extracted from the WoRMS database (https://www.marinespecies.org/).

## Results

### Taxonomic composition

The macrofauna assemblages investigated consisted in total of 16 phyla, 24 classes, 74 orders, and 401 genera. Annelida showed the largest number of taxa, with 463 taxa representing 38.5% of all taxa. This was followed by Mollusca with 233 taxa (19.5% of all taxa) and Arthropoda with 188 taxa (15.6% of all taxa). At the class level, Polychaeta was the most diverse, with 446 taxa, followed by Malacostraca and Bivalvia (Supplementary Table S3). The count data across the 245 samples used for 16S rRNA gene analyses are shown in Supplementary Dataset 1, while the data for the 97 samples used for shotgun sequencing are presented in Supplementary Dataset 2.

For the zOTU data, we identified 7559 archaeal and 112 984 bacterial zOTUs. Combined, these belonged to 132 phyla, with Pseudomonadota being the most diverse, with 28 329 zOTUs, followed by Bacteroidota with 10 930 zOTUs, and Desulfobacterota with 9885 zOTUs (Supplementary Table S4). The percentages of the zOTUs across the 245 samples analyzed are shown in Supplementary Dataset 3.

For the 302 MAGs, we identified 27 phyla, with Pseudomonadota as the most abundant with 106 MAGs, followed by Bacteroidota with 41 MAGs, and Desulfobacterota with 39 MAGs (Supplementary Table S5). The percentages of the MAGs across the 97 analyzed samples are shown in Supplementary Dataset 4.

### Association with metadata

**Figure 3:**
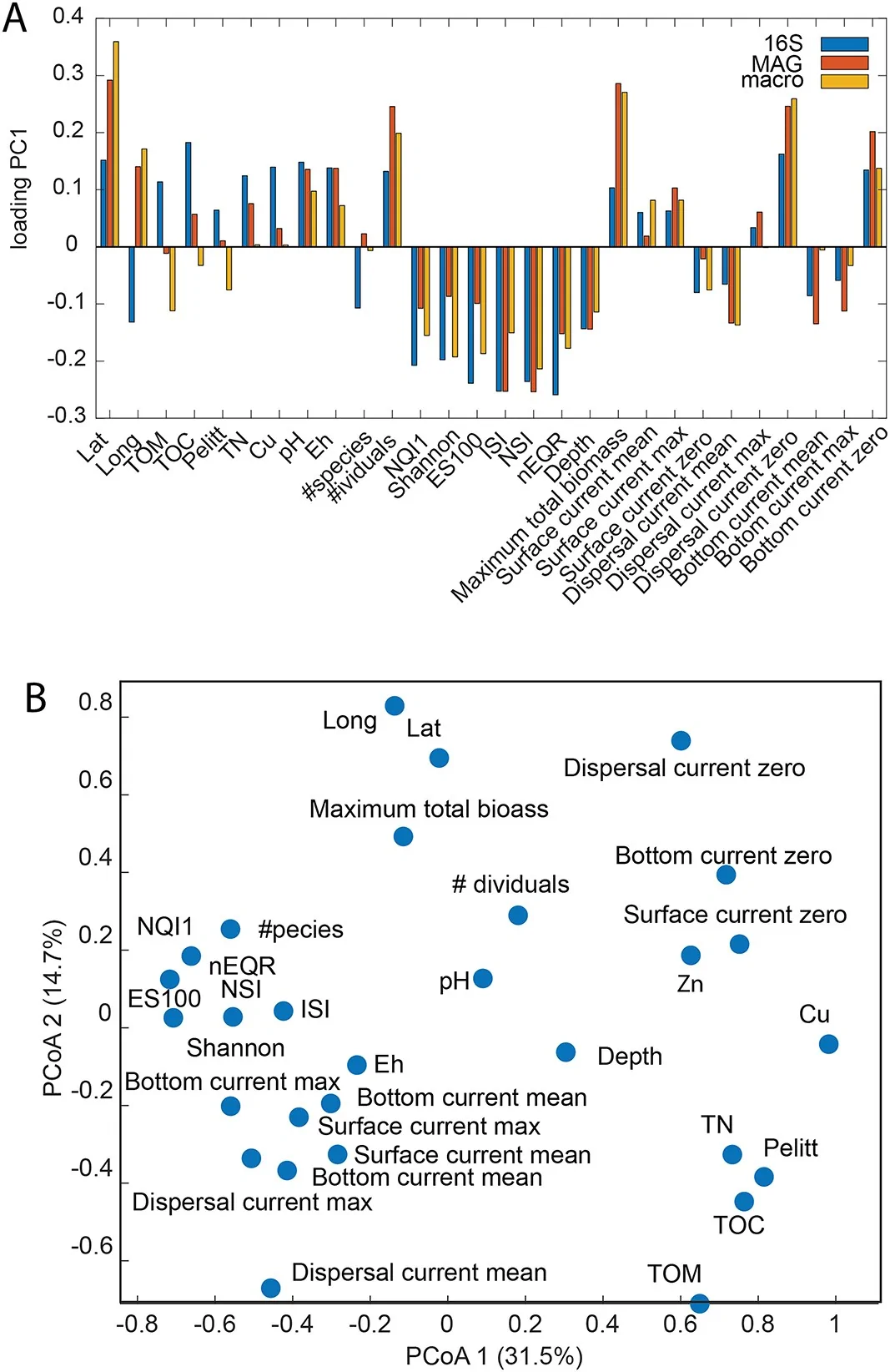
Association of metadata to macrofauna, zOTUs, and MAGs. (A) Association of macrofauna, zOTUs, and MAGs with metadata. The associations were determined through loadings for the first PC. (B) The internal association structure of metadata was determined through PCoA analyses. The first two components are visualized.

Associations between metadata and the datasets (macrofauna, zOTUs, and MAGs) were analyzed using pairwise Spearman correlations. The respective correlation matrices were compressed by PCA analyses. The metadata loadings of the first PCs were subsequently used for dataset comparisons. In these analyses, macrofauna showed the strongest north–south gradient. The ecological indexes showed negative loadings for all three datasets, while maximum total biomass low current showed positive loadings (Fig. 3A). The weakest concordance with metadata was observed between the zOTUs and macrofauna datasets, with Spearman’s rho = 0.76 for the correlation of the metadata loadings for the first PC, whereas the strongest concordance was observed between MAGs and macrofauna, with rho = 0.90. The correlation between MAGs and 16S was intermediate, with rho = 0.83.

The correlation structure across the metadata was analyzed by PCoA. These analyses showed that the ecological index variables were associated with high water current and high redox potential (Eh), which were separated from nitrogen and organic material along the first PC. The second PC appeared to capture geographical differences, with total biomass for production being associated with high longitude and latitude (Fig. 3B).

### Correlation between macrofauna and microbiota

The 16S zOTU and relative MAG abundance datasets were independently correlated to the macrofauna abundance profiles. We identified 6 667 204 positive and 1 489 196 negative correlations between macrofauna taxa and zOTUs for the 16S data (representing 4.6% and 1.0% of the pairwise correlations, respectively), while 10 104 positive and 7566 negative correlations (2.8% and 2.1% of the pairwise correlations, respectively) were identified for the shotgun data (*P* < .05, FDR corrected Spearman correlation for both datasets).

**Figure 4:**
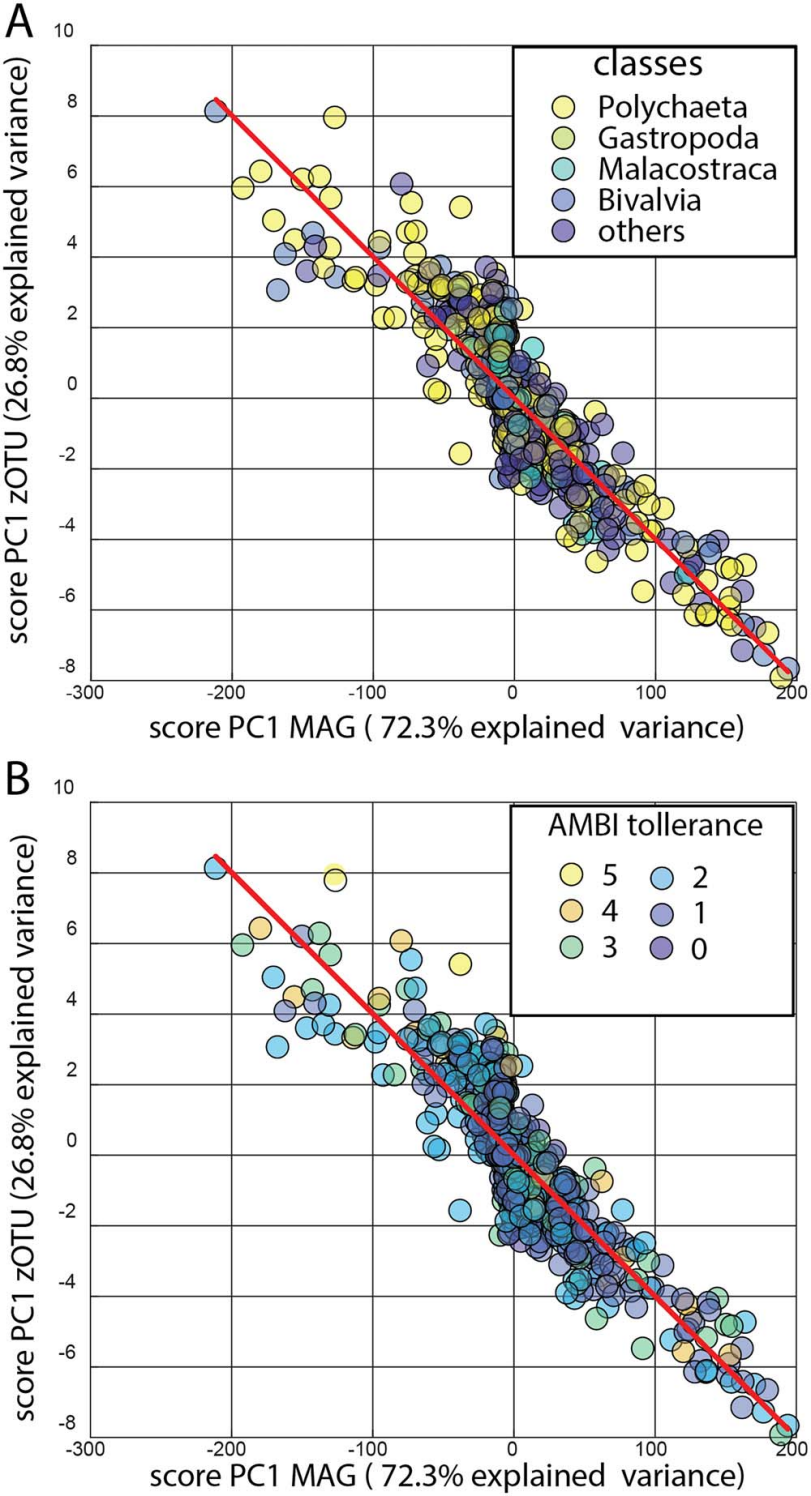
Correlation in scores for the first principal component for macrofauna correlated to MAGs (*y*-axis) and zOTUs (*x*-axis). (A) Color coding presents the class assignments for the macrofauna from the WoRMS database. (B) Color coding presents the AMBI tolerance score. The red lines represent regressions minimizing the squared error.

PCA analyses revealed that 26.8% and 72.3% of the variance could be captured along the first principal components for the 16S and shotgun data, respectively. The comparison of the PC1 scores for the macrofauna from the 16S and shotgun data showed a high correlation (Pearson r = −0.88), indicating that similar macrofauna associations are captured along the first principal component for both the zOTU and MAG datasets; the negative sign reflects the arbitrary orientation of PCA axes (Fig. 4). There was no apparent association between the scores and the taxonomy of the macrofauna (Fig. 4A, *P* > .05, Kruskal–Wallis test), while there was a statistically significant but weak association between AMBI tolerance score and the scores for PC1 (Fig. 4B, Spearman rho = −0.080, *P* = .016 for 16S, and Spearman rho = 0.13, *P* = 1.4 × 10^-4 for MAGs).

### Identification of functional signatures for macrofauna and microbiota associations

**Figure 5:**
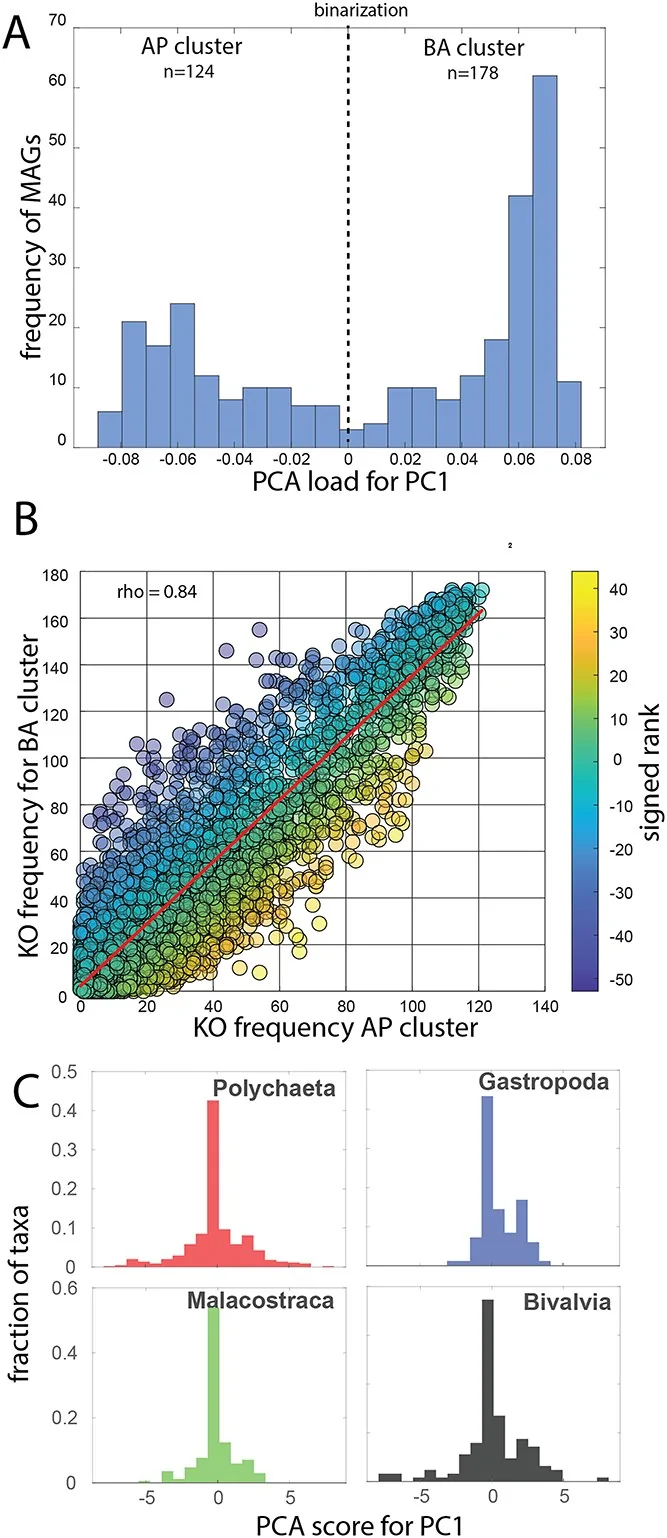
Loadings for the 302 MAGs for the macrofauna correlations. (A) The loadings were split into the BA and AP clusters based on the frequency distribution pattern along the loadings for PC1. (B) For each of the BA and AP clusters, the gene content for the total of 6659 KOs was summed individually, and the deviation was determined for each KO using the signed distance from the regression line representing equal frequency in the BA and AP clusters. Each circle represents one KO with the number of occurrences for the BA cluster along the vertical axis and the occurrences for the AP cluster along the horizontal axis. (C) Macrofauna scores along PC1, histograms highlight frequency of taxa with the strongest positive and negative associations.

The loadings for PC1 for the shotgun data showed a clear bimodal distribution (Fig. 5A, *P* < .001, Silverman’s test). We therefore binarized the data into MAGs showing positive loadings in one group (*n* = 178) and negative loadings in the other (*n* = 124). We also evaluated the threshold for binarization using different cutoffs for the loadings. These analyses indicated similar correlation patterns for loading cutoffs up to ±0.10 (Supplementary Fig. S1).

The loading groups based on a cutoff value of zero showed distinct taxonomic signatures, with Bacteroidota being overrepresented in the cluster with positive loadings (denoted the “BA” cluster), while Actinomycetota and Pseudomonadota were overrepresented in the cluster with negative loadings (denoted the “AP” cluster) (*P* < .05, FDR-corrected Fisher exact test, Supplementary Fig. S2). We observed an overall strong positive correlation for the KOs in the BA and AP clusters. We therefore used the signed perpendicular distance to identify genes that deviated either positively or negatively from the fitted regression line, being selectively overrepresented in the respective groups (Fig. 5B).

For macrofauna, most of the score values were close to zero (Fig. 5C), with only Gastropoda having scores that deviated from unimodality (*P* = .04, Silverman’s test). Macrofauna taxa with high scores (>5) were mainly polychaetes. This group included species such as *Nephtys ciliata*, *Mammiphitime cosmetandra*, *Malacoceros vulgaris*, *Microphthalmus sczelkowii*, *Praxillella praetermissa*, *Spio limicola*, *Ophryotrocha* sp*.*, *Pholoe assimilis*, *Scoloplos armiger*, *Chaetozone setosa*, *Eteone flava/longa*, and *Capitella capitata*. The bivalve *Macoma calcarea* was also found in this group. Taxa with low scores (< −5) were more taxonomically diverse than the high-scoring group. They included many polychaetes, but also several bivalves and ophiuroids. Other groups were represented by a malacostracan, a sipunculan, a copepod, and mollusks (Fig. 5C; Supplementary Table S6).

### Functional associations for genes with signed distance deviation

For the KOs overrepresented in the AP cluster with signed distance >20 (representing 142 KOs, Supplementary Table S7), with the genetic potential for a near-complete NADH dehydrogenase complex I (nuoA/B/C/D/E/G/H/J/K/L/M), in addition to cytochrome bc1 (petC), cytochrome-c biogenesis (ccmA/B/C/E/F/H), and heme-a synthase (ctaA/COX15), were identified. These were identified together with the potential for electron transfer flavoprotein-ubiquinone oxidoreductase (ETFDH/ETF-QO), linking fatty-acid oxidation to the quinone pool. Additional modules include aerobic CO dehydrogenase (coxL/M/S), formate dehydrogenase components (fdhD, fdwB), NADPH:quinone reductase (qor), and the PQQ cofactor pathway (pqqC). KOs representing genes for fatty-acid and branched-chain amino-acid degradation were also enriched (multiple acyl-CoA dehydrogenases including ACADM/fadE, enoyl-CoA hydratase echA, ETFDH, mmsA/mmsB, leucine dehydrogenase), together with matB (malonyl/methylmalonyl-CoA synthetase) and itaconate utilization (ich-Y). Aromatic/organic-acid pathways were also present (pcaD, paaG, hcaC, tcuA, AACS), as were DMSP demethylation genes (dmdB/C), sarcosine oxidation (soxD), nitrile/hydantoin catabolism (nthA, hyuA/B), and dehalogenation of haloalkanes/haloacids (dhaA). Redox homeostasis was centered on glutathione metabolism with the genes gor, gst, gshA, yghU/yfcG, grxD, and CHAC, plus peroxiredoxin (prx3). Additional systems include BolA, HspQ, HrcA, ferredoxin (fdxA), and genes for cofactor biosynthesis for coenzyme F420 (cofC/D/E) and biopterin cycling (PCBD). Together, these overrepresented genes are consistent with genomic potential for oxidative metabolism involving fatty acids, aromatics, amino acids, CO, and DMSP.

The KOs overrepresented in the BA group with signed distance < −20 (representing 226 KOs, Supplementary Table S7) were related carbohydrate acquisition and breakdown genes, including starch/glycogen modules (glgA/B/P), the SusC/SusD outer-membrane complex, multiple glycosidases (β-glucosidase, α-galactosidase, α-L-fucosidases, FUCA), and alginate lyase (alg17C). Central carbon pathway genes were present for glycolysis/gluconeogenesis (pgi, pfkA, fba, gpmI, pgm) and downstream fermentation/short-chain acid formation (ackA/pta, buk). Energy-conservation genes included functions associated with low-oxygen respiration and ion-motive electron transfer, including the Rnf complex (rnfA/B/C/D/E/G), Na + −translocating NADH:quinone oxidoreductase (nqrB/F), high-affinity cytochrome bd oxidase (cydA/B), and multiple ferredoxin-linked oxidoreductases (por/nifJ, kor/oor, iorAB). Sulfur metabolism markers were enriched (sqr; thiosulfate reductase subunit phsC). Hydrogenase maturation genes (hypA-F) were present, while catalytic hydrogenase subunits were not detected. High-affinity phosphate uptake (pstSCAB-phoU) and metal homeostasis modules were enriched: iron uptake and storage (feoAB, ferritin), zinc uptake (znuC, ZIP family), and heavy-metal efflux (cusA/silA, czcA). Stress response and genome maintenance genes include antioxidant systems (peroxiredoxins, thioredoxin, glutathione peroxidase, msrAB, superoxide reductase), chaperones (HtpG, DjlA), toxin-antitoxin components (hipA, yoeB), DNA repair (RecQ, RecJ, MutS2, UDG, RadC), RNA helicases (DeaD/RhlE, HrpA/HrpB), and RNase Y. Overall, the overrepresented genes were consistent with genomic potential for uptake and degradation of complex polysaccharides, nutrient scavenging, metal tolerance, and stress responses under conditions that may include microaerobic or anoxic niches.

### Signed distance difference between BA and AP clusters for enzyme commission categories

**Figure 6:**
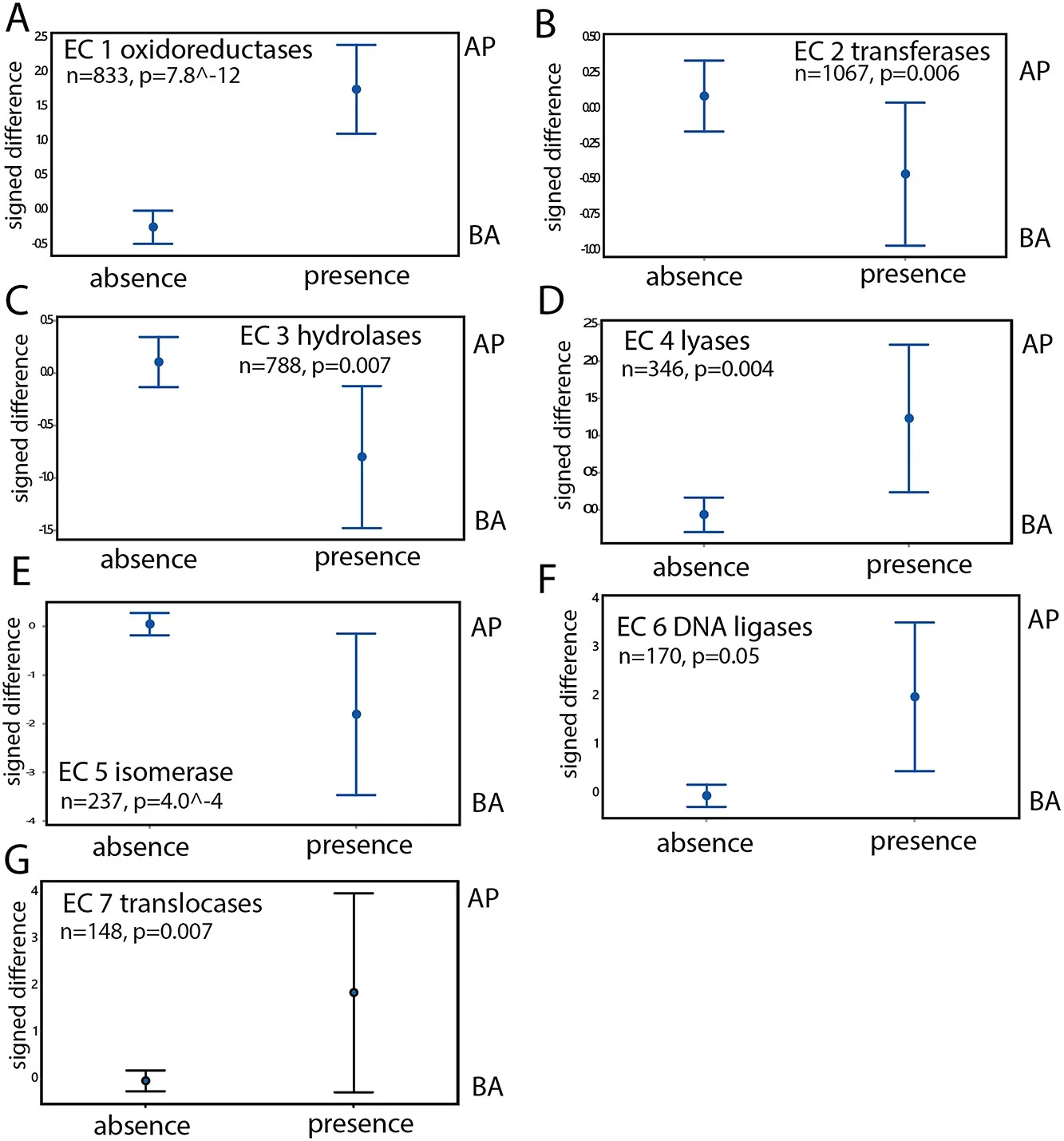
Associations of different EC categories with macrofauna. For each of the seven EC categories, EC1 to EC7 (panels A to G), we determined signed distance differences of genes within the respective EC categories (presence) compared with the rest (absence). Significance was determined using the Kruskal–Wallis test. Mean values and 95% confidence intervals for the mean are shown.

To identify functional differences between the BA and AP clusters at a broader functional level, we investigated whether there was a difference among different enzyme commission (EC) classes. These analyses revealed that oxidoreductases showed the most statistically significant difference for MAGs in the AP cluster (positive signed distance difference), followed by lyases, ligases, and translocases. For the MAGs within the BA cluster (negative signed distance difference), isomerases showed the most statistically significant difference, followed by transferases and hydrolases (Fig. 6).

**Figure 7:**
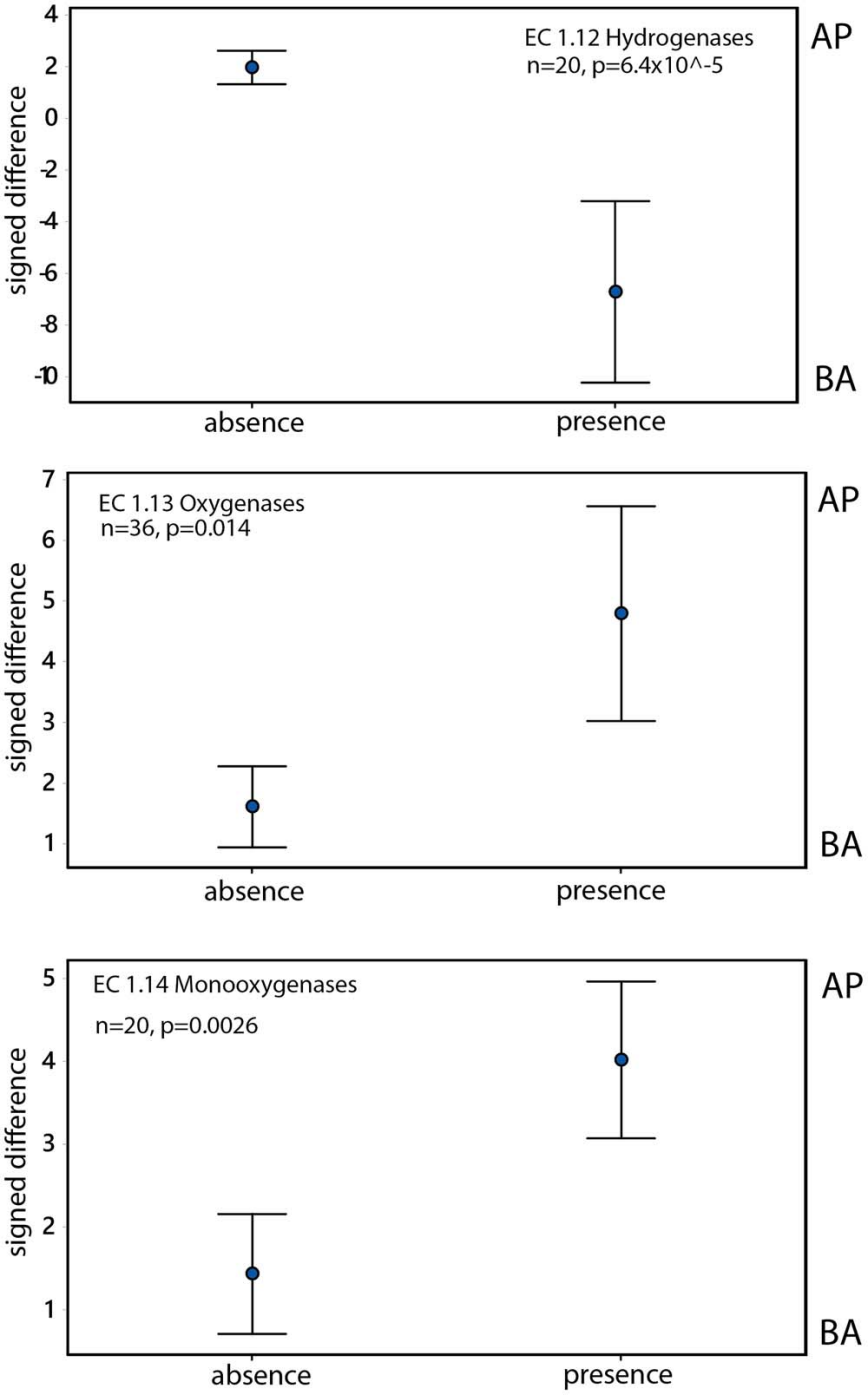
Associations of different EC1 oxidoreductase categories with macrofauna. The three oxidoreductase categories showing statistically significant differences are shown in panels A to C. We determined signed distance differences of genes within the respective EC categories (presence) compared with the rest (absence). Significance was determined using the Kruskal–Wallis test. Mean values and 95% confidence intervals for the mean are shown.

Given the large positive difference for oxidoreductases, we investigated the different classes of oxidoreductases in depth. We identified hydrogenase-related annotations as overrepresented in the BA cluster (negative signed distance difference), while oxidases were overrepresented in the AP cluster as represented by positive signed distance difference (Fig. 7). The other classes of oxidoreductases did not show any statistically significant differences between the clusters after FDR correction.

## Discussion

Whether links between macrofauna and microbiota communities are mainly driven by shared environmental factors, by direct mechanistic interactions, or by feedbacks between macrofaunal activity and microbial processes remains an open question [15–17]. In our work, the stronger north–south gradient in macrofauna suggests greater geographic structure of macrofaunal communities than of microbiota. At the same time, the strong associations of both macrofauna and microbiota with environmental factors are consistent with shared environmental structuring. The microbiota was also strongly associated with ecological indices based on macrofauna composition, suggesting that the two assemblages are jointly informative for assessing ecological state. These patterns are consistent with potential mechanistic links, but they do not establish the direction of causality. A limitation of the present analysis is that oxygen penetration depth and sulfide levels were not available. The metadata analyses, therefore, help identify shared environmental structure, but they cannot fully separate shared environmental responses from direct biotic interactions.

The inferred functional associations of the microbiota with the macrofauna community composition were divided into two distinct clusters for the MAGs, with the BA cluster containing annotations consistent with lower-oxygen metabolism and the AP cluster containing annotations consistent with oxic metabolic processes. The BA-group functional annotation was characterized by genes associated with substrate hydrolysis and microaerobic or anaerobic respiration, whereas the AP-group annotations were characterized by genes associated with oxidase-based substrate decomposition and aerobic respiration.

We found that the BA cluster was overrepresented in deconstruction and import of complex glycans via polysaccharide utilization loci (PULs) through SusC/SusD systems [18]. The BA cluster was enriched in genes encoding Rnf (ferredoxin:NAD+ oxidoreductase) and Na + -NQR, both ion-motive respiratory enzymes supporting growth when external electron acceptors or O2 are limiting [19]. Cytochrome bd is a high-affinity quinol oxidase that can support respiration at low O2 concentrations and resistance to stressors such as sulfide [20]. The annotation for sulfide:quinone oxidoreductase (SQR) identifies a function that can channel electrons from H2S into the quinone pool and has been linked to sulfide detoxification and energy metabolism [21]. Because oxygen penetration, microscale redox conditions, sulfide concentrations, and *in situ* metabolic activity were not directly measured, these environmental and mechanistic interpretations remain inferential. The presence of hydrogenase maturation genes without catalytic hydrogenase subunits suggests possible hydrogenase-related capacity, but not direct evidence for active hydrogen metabolism.

The AP cluster was overrepresented in multicomponent electron transport chains, including complex I, which is linked to the quinone pool through ubiquinol-cytochrome c reductase (CYC1), cytochrome-c biogenesis (Ccm system I), and downstream terminal oxidases. These annotations are consistent with genomic potential for O2 respiration [22]. Electron transfer flavoprotein-ubiquinone oxidoreductase (ETFDH/ETF-QO) is associated with electron transfer from fatty-acid and amino-acid dehydrogenations to the quinone pool and is therefore consistent with potential β-oxidation-linked respiration [23]. The AP cluster also contained PQQ-dependent dehydrogenase annotations associated with the oxidation of small, labile organic compounds. The annotations also indicate genomic potential for DMSP demethylation and aerobic CO oxidation via the cox system [24, 25].

Because our study focused on seafloor microbes, the observed communities likely represent a mixture of free-living environmental taxa and those associated with hosts [26]. However, since we identified only two main microbial clusters linked to macrofauna community composition, the patterns may primarily reflect broad sediment-associated microbial processes rather than highly specific host-associated microbiota, although this interpretation cannot be resolved from the present data. Greater heterogeneity would be expected if the associations were strictly host-associated [27]. Free-living microbes could also be influenced by macrofaunal activity, such as burrowing, feeding, or excretion [15, 17, 28]. In our study, we were not able to disentangle how different macrofaunal activities could influence sediment microbiota composition and function. However, as for the rationale related to host-associated microbes, the two main clusters identify respiratory functional potential as a major axis of covariation.

The AP cluster showed a strong association with gene annotations encoding aerobic carbon monoxide dehydrogenase. One possible interpretation is that this genomic potential could support energy acquisition or detoxification, since carbon monoxide is a strong inhibitor of cytochrome c-based respiration [29]. If these genes are expressed and active, microbial carbon monoxide removal could potentially benefit macrofauna relying on cytochrome c-based respiration [30]. The MAGs within the BA cluster, on the other hand, were enriched in genes annotations associated with polysaccharide and alginate degradation, high-affinity oxygen respiration, and responses to sulfide stress. Because sulfide concentrations and microbial activity were not measured, a contribution of sulfide metabolism to the microbiota-macrofauna association was inferred from the literature, stating that sulfide can affect macrofauna through cytochrome c inhibition under stressful conditions [31].

The BA cluster association with *Capitella, Malacoceros*, and *Ophryotrocha* is consistent with taxa previously associated with high organic loading and reducing conditions, whereas the presence of *M. calcarea* has previously been associated with more transitional conditions [32, 33]. In particular, the genus *Capitella*, which is associated with fermentative microbes, may alter the sediment microbiota [34]. The AP cluster, on the other hand, was associated with higher diversity and less tolerant species [33], while the presence of *Kelliella miliaris* has been reported from an oxygen-minimum zone, and the gills of *K. miliaris* have recently been found to contain high levels of Pseudomonadota, which provides a literature-based explanation for its association with the AP cluster [35]. These ecological interpretations are inferred from taxonomic associations and previous literature rather than from direct measurements of the relevant abiotic conditions. They should also be viewed alongside the weak but statistically significant AMBI-PC1 correlations, which indicate that the tolerance gradient is detectable but not strong.

In conclusion, our work highlights the potential role of respiratory processes in microbiota and macrofauna associations. The results identify clear co-variation between macrofaunal assemblages and microbial respiratory strategies, but do not by themselves resolve causal directionality. We believe this knowledge can be important for the future management of coastal seafloor ecosystems and for designing targeted studies that test how shared environmental gradients, macrofaunal activity, and microbial metabolism interact.

## Supplementary Material

Web_Material_ycag230

## Data Availability

We re-analyzed the raw 16S rRNA and shotgun sequence data available under accession number PRJNA1128851 in the Sequence Read Archive (SRA).
