# Supplementary material for: Contrasting associations between microbiota respiratory strategies and seafloor macrofauna
Source: ISME Commun. 2026 Aug 13;6(1):ycag230. doi: 10.1093/ismeco/ycag230 (PMC13590888; doi:10.1093/ismeco/ycag230)
Supplement: Supplementary file 1 [file web_material_ycag230.zip › SupplFigure1_ycag230.pdf]

A

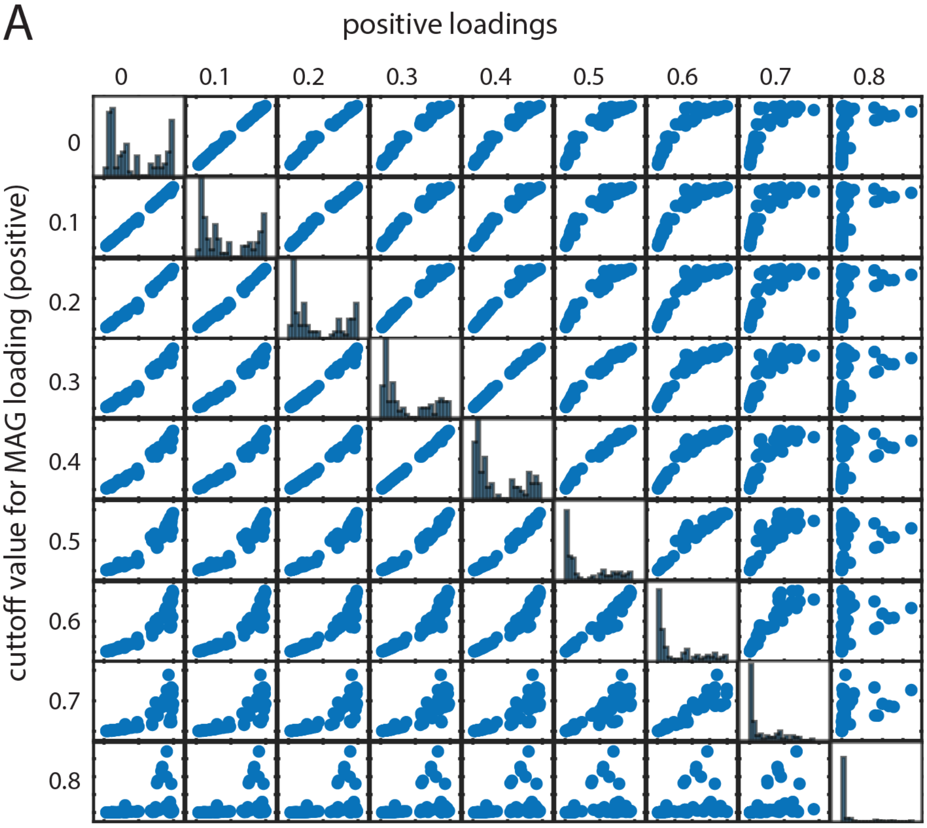

B

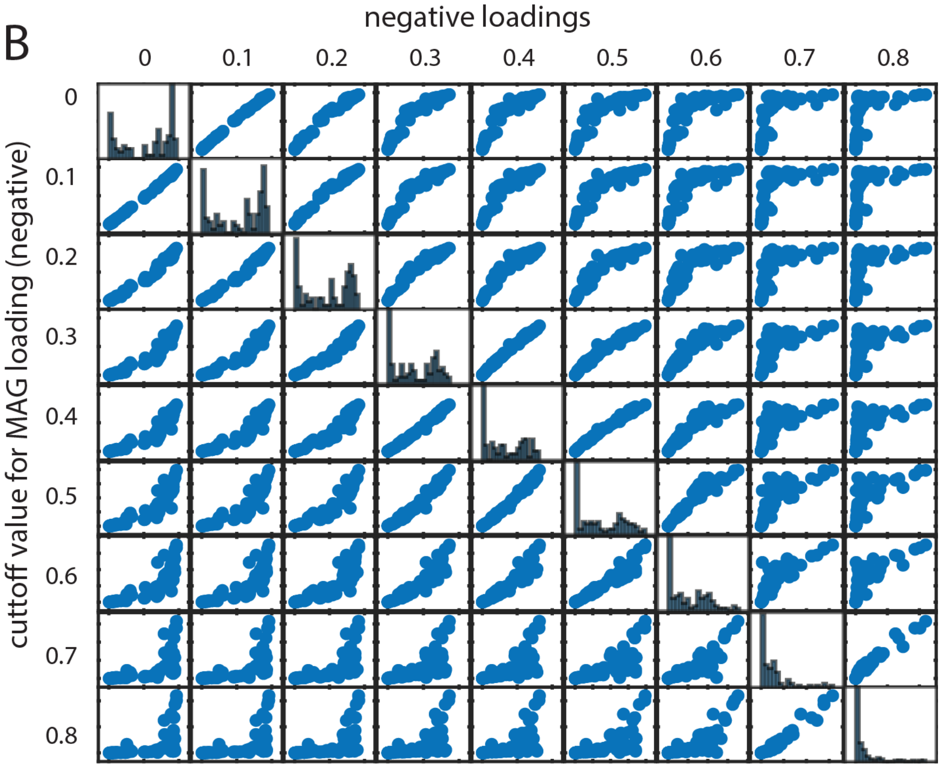

Suppl. Figure 1. Correlation between loading groups with different cutoff values for (A) positive loadings and (B) negative loadings. The loading groups were evaluated by summarizing MAG relative abundance profiles according to model-loading direction and magnitude, as described in Materials and Methods.
