# Supplementary material for: Contrasting associations between microbiota respiratory strategies and seafloor macrofauna
Source: ISME Commun. 2026 Aug 13;6(1):ycag230. doi: 10.1093/ismeco/ycag230 (PMC13590888; doi:10.1093/ismeco/ycag230)
Supplement: Supplementary file 1 [file web_material_ycag230.zip › SupplFigure2_ycag230.pdf]

## SUPPLEMENTARY FIGURES

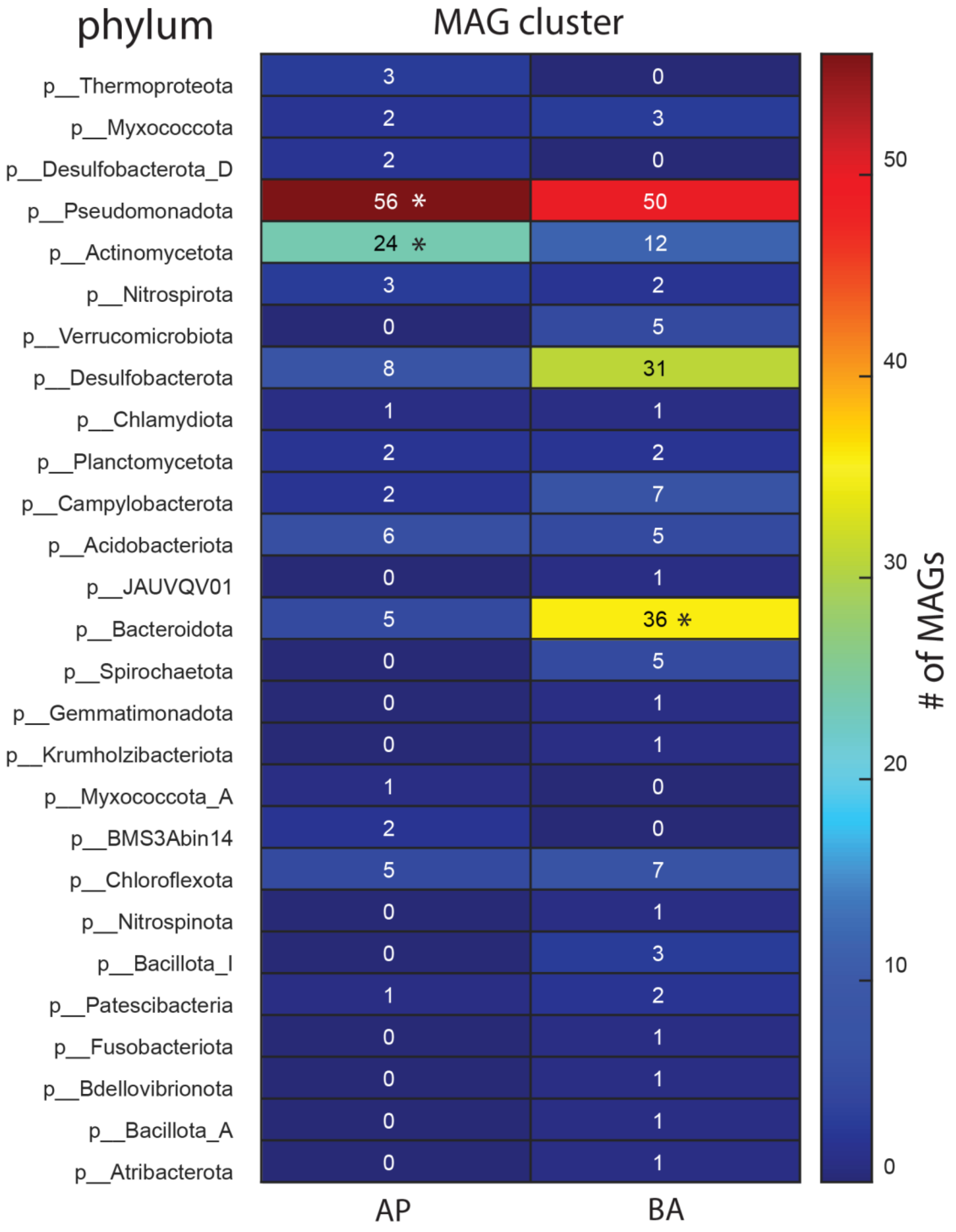


**Suppl. Figure 2. Phylum distribution of MAGs between the AP and BA cluster.** The heatmap presents the number MAGs classified in the different phylum categories for the AP and BA clusters. The phyla that showed statistically significant differences (Fisher exact test < 0.05, FDR corrected) are marked with asterisks.
